# Genetic Diversity and Population Structure of Wild Ancient *Camellia tetracocca* in Pu’an, Guizhou, China

**DOI:** 10.3390/plants14111709

**Published:** 2025-06-04

**Authors:** Deqin Li, Lushan Li, Shukui Chang, Shunrong Zhang, Jian Feng, Lifei Wang, Xiaoxia Huang, Huizhen Hu, Feng Zu, Xiaomao Cheng

**Affiliations:** 1 Engineering Technology Research Center of National Forestry and Grassland Administration on Southwest Landscape Architecture, College of Landscape Architecture and Horticulture Sciences, Southwest Forestry University, Kunming 650224, China; m18212581442@163.com (D.L.); 18086945551@163.com (L.L.); yndl15368307813@163.com (S.C.); zsr13888727462@163.com (S.Z.); hclyj2854@163.com (L.W.); huangxx@swfu.edu.cn (X.H.); jenny_0129@swfu.edu.cn (H.H.); 2 Yunnan Key Laboratory of Genetic Improvement of Herbal Oil Crops, Industrial Crops Research Institute, Yunnan Academy of Agricultural Sciences, Kunming 650225, China; 3 Zhenyuan Yi Hani Lahu Autonomous County Forestry Science and Technology Extension Center, Lincang 666599, China; fengjian311@126.com

**Keywords:** *Camellia tetracocca*, wild ancient tea plant, genetic diversity, population structure, intron-length polymorphism markers

## Abstract

Pu’an County, located in southwestern Guizhou Province, China, is one of the original habitats for wild tea plants. It is renowned not only as the “Home of Ancient Tea Trees in China” but also as the “Core Production Area for High-Quality Early Tea in China”. The wild ancient *Camellia tetracocca* tea trees are considered “living fossil”. Understanding the genetic diversity of wild ancient *C. tetracocca* in Pu’an, Guizhou, is of great significance for addressing conservation concerns and mitigating genetic erosion in this endemic species. This study investigates the genetic diversity and population structure of wild ancient *C. tetracocca* tea plants in Pu’an County to support the development of conservation strategies. We genotyped 138 ancient wild *C. tetracocca* specimens using 40 intron-length polymorphism markers. A total of 180 alleles were detected, with the allele numbers per locus ranging from 2 to 10 and an average of 4.50. The number of effective alleles varied from 1.36 to 8.01, with an average of 2.86. The Shannon information index ranged from 0.28 to 2.19, with an average of 1.10. Nei’s gene diversity index ranged from 0.14 to 0.88, with an average of 0.58. The polymorphic information content (PIC) varied from 0.14 to 0.85, with an average of 0.58. Our findings indicate that the genetic diversity of wild ancient *C. tetracocca* tea plants in Pu’an is high. Specifically, the genetic diversity in Qingshan Township surpassed that in Xindian Township. Analysis of molecular variance indicated that 91.59% of the genetic variation occurred within the subpopulations, suggesting limited differentiation. Despite their geographical separation, populations from Qingshan and Xindian showed a complex genetic relationship (*F*_ST_ = 0.04). STRUCTURE analysis identified three distinct genetic clusters, indicating a complex demographic history. These findings underscore the conservation significance of wild *C. tetracocca* populations in Pu’an and highlight the need for conservation strategies that prioritize the protection of genetically diverse subpopulations, especially in the Qingshan region.

## 1. Introduction

*Camellia tetracocca* is a perennial wild tea plant that belongs to the genus *Camellia* L., subgenus Thea, series Quiquelocularis [1]. This rare and unique species is endemic to Pu’an County, Guizhou Province, China, and is often referred to as the ‘drinkable living fossil’. It primarily grows in Majiaping Village, Pubai Forest Farm, and other mountainous and forested areas at elevations ranging from 1700 to 1950 m above sea level in Pu’an County, in southwest Guizhou Province. The population comprises wild, cultivated, and transitional types that exhibit distinct characteristics influenced by the growing environment [2,3,4]. Significant morphological differences were observed among wild, transitional, and cultivated tea plants of Puan’s *C. tetracocca*, which stemmed from factors such as the growing environment, level of human intervention, and tree age. Wild tea trees are typically tall and upright, with a prominent main trunk. Their posture is either erect or semi-spreading, with fewer and irregular branches. After pruning, transitional tea plants tend to have a broader canopy, more branches, and clearer layering, with a shortened main trunk. In contrast, cultivated tea plants, owing to dense planting and pruning techniques, grow in a bushy form, with a low and dense canopy and no distinct main trunk. The discovery of *C. tetracocca* seed fossil in Yuntou Mountain, Pu’an County, dating back over one million years, indicates its long history in southwest Guizhou and highlights its significant historical and cultural value [2]. The leaves of *C. tetracocca*, similar to those of the cultivated tea plants (*C. sinensis*), are used by locals to prepare tea, a popular non-alcoholic beverage known for its health benefits [5]. Owing to its primitive growth environment and slow growth rate, *C. tetracocca* is characterized by low yield and high quality, making it ideal for producing premium black tea [6]. The limited and distinct distribution of tea means that it is currently cultivated and consumed exclusively in a local area [7]. Although tea production has notably boosted local incomes, its wild habitat has been severely affected by over-harvesting, environmental degradation, large-scale pest outbreaks, and plant diseases [8]. *C. tetracocca*’s unique genetic composition and chemical characteristics make it a valuable resource for developing new tea cultivars with enhanced flavors and health benefits [9]. The presence of specific secondary metabolites, such as catechins and theacrine [5], presents opportunities for breeding tea plants that possess superior antioxidant properties and lower caffeine content in response to the growing consumer demand for healthier tea options [6].

In recent years, research has increasingly focused on exploring the genetic diversity of tea germplasms using polymorphic DNA-based techniques, with molecular markers emerging as the most effective methods. Molecular markers offer significant advantages by directly detecting variations in DNA, thereby avoiding interference from environmental factors such as temperature, light, and nutrition, as well as phenotypic traits. Owing to their high precision, throughput, and objectivity, molecular markers have become essential tools for studying plant genetic diversity [10]. Advances in molecular marker technologies have significantly enhanced several research fields, including the differentiation of plant varieties and cultivars, the assessment of genetic stability, the analysis of genetic diversity and relationships, and the construction of genetic maps [11]. Various molecular markers, such as inter-simple sequence repeats (ISSR) [12], amplified fragment length polymorphism (AFLP) [13], single nucleotide polymorphisms (SNP) [14,15,16,17,18,19], and microsatellites or simple sequence repeats (SSR) [20,21,22,23], have been employed to assess genetic diversity, relationships, and construct genetic maps in tea plants. These studies demonstrate that molecular markers can effectively authenticate tea plant varieties and clarify their genetic relationships [12,13,14,22]. It is widely believed that tea plants originated in the southwestern region of Yunnan, China [13,16]. Furthermore, tea plants from regions such as Yunnan, Guizhou, Sichuan, and Fujian exhibit considerable genetic diversity, making these areas vital for resource conservation and utilization [13,16,17,18,19,20,24]. Additionally, significant genetic differentiation has been observed between wild and cultivated tea plants [13,16]. These methods provide valuable tools for the comprehensive evaluation of genetic variation within tea germplasm resources. Among these techniques, Intron Length Polymorphism (ILP) has gained particular recognition as a vital tool in tea plant genetics and breeding, owing to its high abundance, substantial polymorphism, reliability, and cross-species transferability [24,25]. Molecular marker technologies have revealed that wild ancient tea trees possess considerable genetic diversity, with distinct geographic differentiation observed in the genetic structure of populations across different regions [17,26,27,28]. These discoveries provide critical scientific data for the conservation and rational utilization of wild ancient tea tree resources. Furthermore, the genetic diversity observed in ancient tea trees results not only from natural evolutionary processes but also reflects the influence of domestication and modern breeding practices. The identification of domestication and breeding sweeps within the tea plant genome highlights the selective pressures shaping the genetic composition of current tea cultivars. These findings are essential for understanding the genetic basis of key agronomic traits and for developing future breeding strategies [29].

Molecular markers have played a pivotal role in revealing the extensive genetic diversity of wild ancient tea trees. Genetic variability is a crucial resource for the conservation and enhancement of tea plants, contributing to their resilience and adaptability to environmental changes and evolving agricultural demands [30]. In the present study, we used 40 pairs of ILP molecular markers to investigate the genetic diversity and population structure of 138 wild ancient *C. tetracocca* tea trees from two townships in Pu’an County, Guizhou Province, China. The primary goal of this study was to gain a deeper understanding of the genetic traits of wild ancient *C. tetracocca* in this area and provide essential scientific insights and baseline data to support its development, conservation, and molecular marker-assisted breeding.

## 2. Results

### 2.1. Evaluation of Genetic Parameters and the Degree of Diversity at ILP Loci

Genetic diversity analysis was conducted on 138 wild-type ancient *C. tetracocca* tea plants collected from Qingshan and Xindian towns in Pu’an County (Table 1). The results revealed 180 observed alleles (Na) across 40 loci. Na per ILP locus ranging from 2 to 10, with an average of 4.56. The effective allele numbers (Ne) ranged from 1.16 to 8.01, with the lowest value observed at Tea_ILP3087 and the highest at Tea_ILP1986, yielding an average Ne of 2.86. Observed heterozygosity (Ho) varied from 0.00 to 0.89, with an average of 0.20, while expected heterozygosity (He) varied from 0.14 to 0.88, with a mean of 0.58. Shannon’s information index (I) ranged from 0.28 to 2.19, with the lowest value at Tea_ILP3087 and the highest at locus Tea_ILP1986, with an average of 1.10. Similarly, Nei’s gene diversity index (H) ranged from 0.14 to 0.88, with the smallest and largest values observed for Tea_ILP3087 and Tea_ILP1986, respectively, yielding a mean of 0.58. Polymorphic Information Content (PIC) ranged from 0.14 to 0.85, with a mean of 0.58. Notably, the locus Tea_ILP3087 was the only site with a polymorphism below 0.25, categorized as low polymorphism, whereas the remaining loci exhibited medium to high polymorphism levels, with a mean of 59.26%.

### 2.2. Evaluation of the Genetic Diversity Level of Wild Ancient Tea Populations in Pu’an

Genetic diversity analysis was conducted on wild-type ancient *C. tetracocca* populations from two locations in Pu’an, Qingshan Town, and Xindian Town (Table 2). Na for the two populations were 4.38 and 4.05, respectively. The Ne values were 2.98 and 2.30, respectively. Both populations exhibited a heterozygosity (Ho) value of 0.20, while the expected heterozygosity (He) values were 0.60 and 0.50, respectively. Additionally, the values for Shannon’s index (I) were 1.13 and 0.90, and the values for Nei’s genetic diversity index (H) were 0.60 and 0.50, respectively. The wild ancient tea populations of Qingshan Town and Xindian Town exhibited significant differences (*p* < 0.05) in genetic diversity parameters, including I, He, H, and A_R_.

### 2.3. Genetic Relationship and Genetic Differentiation of Wild Ancient C. tetracocca Population in Pu’an

The genetic similarity coefficient between the wild ancient *C. tetracocca* populations in Qingshan and Xindian towns was 0.92, whereas the genetic distance was 0.09. The genetic relationship between these two populations in Pu’an County was relatively close, likely because of the frequent gene flow between the wild tea trees in both regions, which led to a high degree of genetic similarity.

Genetic differentiation among populations was analyzed using POPGENE, which revealed an inbreeding coefficient (F_is_) of 0.64 within populations and a total population inbreeding coefficient (F_it_) of 0.65. The population differentiation coefficient (F_st_) was 0.04, indicating that the genetic variability in the wild-type ancient *C. tetracocca* populations was primarily within populations. Gene flow (N_m_) between populations was 6.85, suggesting substantial gene exchange. Furthermore, AMOVA analysis showed that 8.41% of the genetic variation was attributed to inter-population differences, while a significant 91.59% occurred within populations (Table 3).

### 2.4. Genetic Structure of Wild Ancient C. tetracocca Population in Pu’an

**Figure 1:**
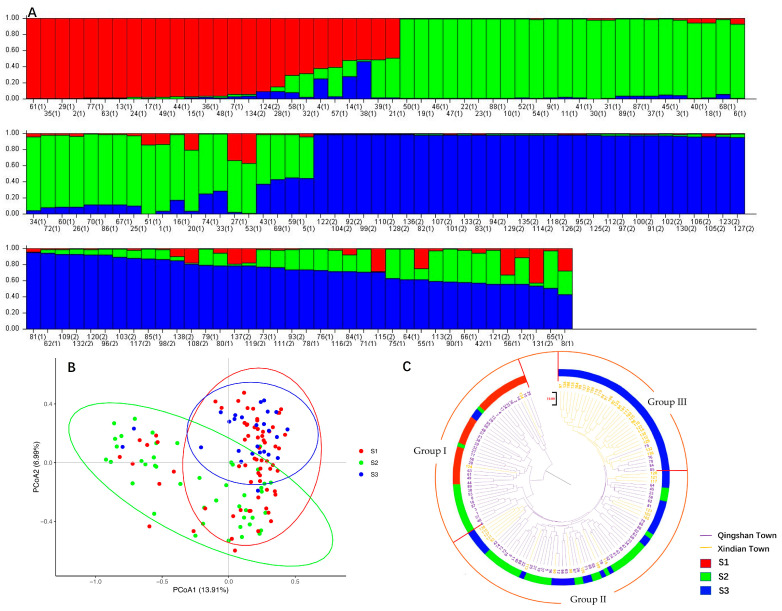
Distribution of Q values for the population structure of wild ancient *C. tetracocca* in Pu’an. (**A**) A total of 138 individuals were categorized into three subpopulations: S1 (red bar), S2 (green bar), and S3 (blue bar), consisting of 26, 44, and 68 individuals, respectively. The samples are numbered from 1 to 138, where (1) corresponds to the population from Qingshan Township and (2) represents the population from Xindian Township. (**B**) Principal component analysis identified three inferred populations in STRUCTURE, with S1 shown in red, S2 in green, and S3 in blue. (**C**) Phylogenetic tree of 138 wild ancient tea germplasms and the three inferred populations: Group I, Group II, and Group III. Samples 1–90 correspond to the population from Qingshan Town, and samples 91–138 correspond to the population from Xindian Town.

Based on this analysis, the optimal number of genetic clusters was determined to be K = 3, representing the most biologically meaningful subdivision of *C. tetracocca* populations (Figure 1A). These three subpopulations exhibited distinct genetic structures, as illustrated in the STRUCTURE bar plot. The S1 cluster (red bar) comprised 26 samples, 24 of which (92%) were wild ancient *C. tetracocca* individuals from the town of Qingshan. The S2 cluster (green bar) consists exclusively of 44 wild ancient *C. tetracocca* samples from Qingshan Town. In contrast, the S3 cluster (blue bar) includes 68 samples: 22 (32%) from Qingshan Town and 46 (68%) from Xindian Town. This uneven distribution highlights significant geographic differentiation, with Qingshan Town contributing disproportionately to both the homogeneous (S2) and admixed (S3) clusters, whereas Xindian Town was represented solely within S3.

Principal component analysis (PCoA) was applied to analyze the wild ancient *C. tetracocca* population in Pu’an (Figure 1B). The analysis revealed a distinct clustering of wild-type ancient tea trees within individual regions, with partial overlaps between clusters. These findings are consistent with the results from the phylogenetic and STRUCTURE analyses, which similarly identified heightened genetic affinity between the Qingshan and Xindian populations (Figure 1A,C).

A hierarchical clustering tree was constructed using MEGA11 (v. 11.0.13), which classified the 138 ancient wild tea plant germplasms into three distinct groups (Figure 1C). Group I comprised 41 ancient wild tea germplasms, 39 of which were from Qingshan Town. Group II comprised 62 germplasm lines, including 15 from Xindian Town and 47 from Qingshan Town. Group III consisted of 35 germplasms, predominantly from Xindian, with only 5 germplasms from Qingshan. Notably, genotypic crossovers were primarily observed in the germplasm resources of Qingshan Town and Xindian Town in Group II, indicating genetic exchange between these populations. This finding aligns with those of the population structure and PCoA analyses (Figure 1A,B). Additionally, the clustering map indicated that although ancient wild tea plant germplasms from Qingshan Town and Xindian Town showed some overlap, they maintained distinct genetic characteristics.

## 3. Discussion

### 3.1. Genetic Diversity of Wild Ancient Tea Plants of C. tetracocca Using ILP Markers

The recent availability of high-quality tea plant genomes, including Yunkang10 [31], Shuchazao [32], wild tea accessions [28], Longjing43 [33], Tieguanyin [34], Biyun [35], and Huangdan [36], has led to significantly advanced functional and comparative genomic studies [15,16]. The tea plant genome is notable for its high intron density, which provides considerable advantages for species identification and the development of molecular markers [25]. ILP markers have emerged as a powerful tool for evaluating genetic diversity in *C. sinensis*, demonstrating superior effectiveness compared to traditional markers such as SSRs and SNPs [25]. ILP markers target polymorphic regions within intron non-coding sequences that exhibit higher mutation rates than exons, owing to relaxed selective pressures. This variability makes ILPs particularly useful for detecting subtle genetic differences among closely related tea cultivars, allowing them to effectively capture intra-specific diversity [28]. However, for non-model organisms lacking genomic information, the detection cost and technical barriers for ILP markers remain relatively high. Although introns are non-coding regions that may participate in the regulation of gene expression, some ILP markers are influenced by natural selection. This contradicts the neutral evolution hypothesis, resulting in analysis outcomes confounded by population history and selective pressures.

In this study, we analyzed the genetic diversity and relationships of 138 ancient wild tea plants collected from Qingshan and Xindian Towns in Pu’an County using 40 pairs of ILP loci. The He value is a key indicator of a marker’s utility for cultivar identification, as it reflects the diversity within a species [37]. A higher He value indicates that the marker is more informative and reveals greater genetic variation among sampled individuals [38]. In contrast, Ho represents the probability that the alleles of two randomly selected samples differ. The results for Ho and He across the 40 pairs of ILP loci in this study (Table 1) show that Ho ranges from 0.00 to 0.89, with an average of 0.20, while He ranges from 0.14 to 0.88, with an average of 0.58. The observed deficit in heterozygosity (Ho < He) in wild ancient *C. tetracocca* populations in Pu’an suggests notable genetic constraints and population substructures. Such patterns are often linked to inbreeding, Wahlund effects caused by undetected subpopulations, or genetic drift in fragmented habitats.

The PIC value, which measures locus polymorphism [39], is useful for assessing genetic diversity [40]. In this study, PIC values ranged from 0.14 to 0.85, with an average of 0.58. Among the loci analyzed, 29 displayed high polymorphism (PIC ≥ 0.5), 10 exhibited moderate polymorphism (0.25 ≤ PIC < 0.5), and one showed low polymorphism (PIC < 0.25). These findings suggested that the primers generally exhibited strong polymorphism [41]. The mean PIC value for all ILP markers was 0.58, indicating a high level of genetic diversity within wild ancient *C. tetracocca*, which aligns with the findings of previous studies [4]. However, it was lower than those reported by [42] (0.86) and [23] (0.69) yet higher than the values observed by [20] (0.45) and [25] (0.48). The degree of polymorphism is related to primer discrimination; a higher degree of polymorphism enhances primer discrimination. Moreover, comparisons should be made with caution due to differences in sample size, marker type and number, and the specific statistical approaches used to estimate genetic diversity.

Life-history traits and geographic factors play a crucial role in shaping genetic diversity [43]. Species with restricted distributions generally exhibit lower genetic diversity than those with broader distributions [26,44], often because of smaller population sizes and adaptation to specific habitats resulting from isolation [45]. Although the effects of habitat fragmentation on population genetics have been studied for several decades, clear response patterns remain elusive [46]. Previous research has indicated that favorable environmental conditions promote gene flow among plant populations, thereby enhancing genetic diversity. For instance, plant populations at mid-altitudes often display higher genetic diversity than those at lower or higher altitudes, probably because of the more favorable environmental conditions at these elevations [46,47]. In the present study, the genetic diversity indices (I = 1.13, H = 0.60) for the Qingshan Town population were significantly higher than those for the Xindian Town population (I = 0.90, H = 0.50), suggesting greater genetic diversity in the Qingshan Town population. A plausible explanation is that, despite being a restricted-range and endangered endemic species, *C. tetracocca* thrives at the moderate elevation of Qingshan Town (1699–1744 m), which provides more favorable temperature and soil conditions than Xindian Town (1822–1834 m), thereby promoting population growth [28]. Additionally, the self-incompatibility of tea plants fosters genetic diversity, enriching the genetic pool and enhancing the species’ ability to adapt to environmental changes.

### 3.2. Molecular Variation and Population Genetic Structure

High-frequency alleles from plants bred outdoors, such as cross-pollinated wind-borne species, were observed in each population, showing high similarity and minimal genetic variation [48]. AMOVA in this study revealed that 8.41% of the genetic variation occurred among populations, whereas 91.59% occurred within populations (Table 2), which is consistent with the characteristics of a wild outcrossing species. This indicates significant gene flow between populations (*N*_m_ = 6.85). Similar results were found in SSR analyses of the ancient tea germplasm of Sandu County, Guizhou Province, China, which indicated that 95% of genetic diversity resides within populations [49]). Similarly, SSR analysis of Korean cultivated tea germplasm indicated that 99% of the genetic diversity was within the populations [50]. The tea plant, a long-lived woody species with high rates of cross-pollination, facilitates gene exchange across populations, resulting in low genetic differentiation.

**Figure 2:**
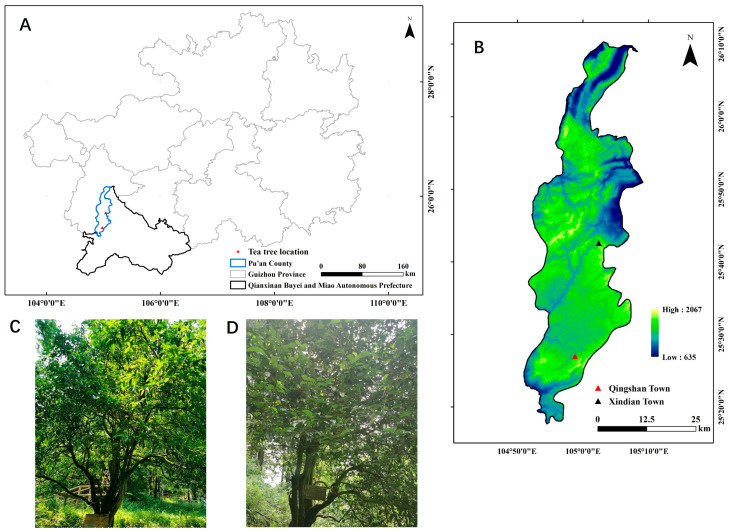
Qingshan Town and Xindian Town are geographically close, located in the south of Pu’an County, Guizhou Province. (**A**) shows the location map of Puan County in the Qiannan Buyei and Miao Autonomous Prefecture, Guizhou Province, China. (**B**) shows the locations of the sampling sites, Qingshan Town and Xindian Town, within Puan County. (**C**,**D**) are photos of individual plants from the Qingshan Town and Xindian Town populations, respectively.

Genetic structure analysis, including PCoA and phylogenetic cluster analysis, revealed that the two wild ancient tea populations could be grouped into three clusters. We propose two explanations for this classification. First, environmental factors—such as geology, altitude, temperature, rainfall, and light—significantly affect tea plant growth. Specifically, altitude and geological conditions play crucial roles in the distribution, population structure, and evolutionary trajectory of wild plant germplasm [51,52]. Geographical distance between tea populations is often highly correlated with genetic distance [7,15], and local populations in geographically contiguous areas tend to share closer genetic relationships [23,53]. Qingshan Town and Xindian Town are geographically close and are situated in the southern part of Pu’an County, Guizhou Province (Figure 2). Genetic differentiation and consistency analyses of the two populations showed minimal differentiation and high genetic consistency, confirming the close genetic relationship between local populations in nearby geographical areas. The second factor influencing gene exchange is geographical proximity, which likely facilitates gene flow among populations. Zhao et al. [54] classified tea plants cultivated in the Guizhou Plateau into four groups based on river basin distributions. In this study, Qingshan Town and Xindian Town, located approximately 70 km apart in the northern part of Pu’an County (Figure 2), showed a high potential for gene flow because of their proximity. Genetic differentiation and consistency analyses of the two populations revealed minimal differentiation, suggesting that gene flow played a significant role in reducing genetic differentiation. When *N*_m_ is greater than one, a sufficient gene flow exists to counteract genetic drift, which typically leads to population divergence [47]. The high gene flow observed in this study (Nm = 6.85) likely explains the low genetic differentiation found among the wild ancient tea populations. This can be attributed to several factors, including the wind-pollinated nature of *C. tetracocca*, which allows it to travel over long distances. Additionally, the unique biological traits of the species, such as monoecy, high outcrossing rates, long lifespans, and overlapping generations, contribute to the reduced genetic differentiation between populations.

### 3.3. Wild Ancient Tea Germplasm Conservation and Utilization

The study of genetic diversity within species and the genetic structure of populations is crucial for the development and implementation of effective conservation strategies [55]. This study revealed that the wild ancient populations of the endemic species *C. tetracocca* in Pu’an exhibited relatively high genetic diversity, positioning them as a key germplasm resource for tea plant genetic improvement. The findings underscore the urgent need for conservation measures in light of the increasing demand for raw tea materials and the unsustainable pressures faced by these populations due to over-exploitation [56,57]. Furthermore, non-selective deforestation and habitat destruction driven by agricultural expansion exacerbates survival threats to these wild populations. As the market demand for organic tea products increases, the over-harvesting of tea trees by local communities has become more pronounced. Additionally, poorly planned harvesting and plant transplantation strategies have resulted in the irreversible loss of valuable genetic resources [4].

Biotic stress factors, such as pests and diseases, combined with abiotic stressors, such as climate change, further underscore the importance of conserving the germplasm of wild ancient tea tree species. This conservation effort is essential not only for maintaining ecological resilience but also for ensuring the sustainable development of the tea industry. In response to these challenges, it is crucial to establish an ecological compensation mechanism to safeguard the interests of the stakeholders involved in the conservation of wild ancient tea tree populations [22]. Furthermore, the biological characteristics of *C. tetracocca* enhance its conservation potential. This species exhibits remarkable cold tolerance (≤6.0 °C), and its genome offers valuable resources for distant hybridization and gene introgression breeding, positioning it as key genetic material for developing breakthrough tea tree varieties [4,58].

Wild resources of *C. tetracocca* are rare and of exceptional quality. Therefore, it is crucial to conserve these resources by covering as many types of germplasm as possible to maximize genetic diversity [43]. In addition to in situ conservation, we recommend the following measures: (1) establishing a germplasm resource bank for *C. tetracocca*; (2) developing and promoting artificial cultivation methods; (3) enhancing scientific research on the species, particularly in the fields of ecology, genetics, and reproductive biology; (4) raising awareness among local communities and the general public about the protection of wild *C. tetracocca*, encouraging their active participation in conservation efforts. These actions support the long-term survival and development of *C. tetracocca*, a species endemic to Guizhou while preserving its genetic diversity through a comprehensive, multi-tiered conservation approach based on in situ conservation. Niu et al. [27] noted that the Guizhou Plateau is rich in ancient local varieties and pure wild germplasm resources, which form an important foundation for tea tree breeding programs. Wild tea tree populations of *C. tetracocca* have significant scientific value in the conservation and research of tea tree genetic resources. Using single-plant breeding methods, new tea tree varieties can be developed from these germplasm resources, which are both practical for production and valuable genetic parent material.

## 4. Materials and Methods

### 4.1. Plant Materials

This study focused on wild ancient *C. tetracocca* located across Pu’an County, Guizhou Province (Figure 2). The distribution area of the wild ancient *C. tetracocca* is very narrow, primarily concentrated on the mountain slopes and forest edges of the Pu’bai Forest Farm in Qingshan Town, within an elevation range of 1700–1950 m [2], with a small distribution in other towns, such as Xindian Town. The total distribution area is less than 60 km^2^, and it is an endemic species of Pu’an County, with the type specimen collected in the Pu’bai Forest Farm. The climate of this region is subtropical humid monsoon. The average annual temperature is 14 °C, with an extreme minimum temperature of −6.9 °C and an extreme maximum temperature of 33.4 °C. The frost-free period lasted for 297 days, with abundant rainfall, an annual precipitation of 1257 mm, and high humidity, with a relative humidity of over 80%. The average annual sunshine duration is 1287 h. In total, 138 specimens were collected for experimental analysis, with 90 wild-type *C. tetracocca* trees sampled from Qingshan Town and 48 from Xindian Town, both within the county. Upon collection, leaf samples were immediately placed in self-sealing bags with color-changing silica gel for drying and preliminary processing. Appropriate labels were simultaneously affixed to the samples, which were then stored at room temperature. Detailed characteristics and information regarding each tea plant variety are provided in Table 4.

### 4.2. ILP Primer Sources

We amplified six randomly selected samples of *C. tetracocca* DNA templates using 230 ILP primer pairs and obtained 40 primer pairs with abundant polymorphism and clear amplification bands for genetic diversity analysis of wild ancient tea trees [25]. Detailed information on these 40 primer pairs is provided in Table 5.

### 4.3. DNA Extraction, PCR Amplification, and Product Detection

DNA was extracted from Tetrahymena using a modified CTAB method, following the protocol described by Tang et al. [59]. The PCR reaction system and procedure were based on the method outlined by Jayaswall et al. [60] with a total volume of 10 µL. The PCR mixture consisted of 2 µL of DNA template (50 ng/µL), 0.5 µL of primer (10 µmol/µL), and 7.5 µL of 2× Taq master mix. After gentle mixing, the mixture was briefly centrifuged for 30 s and sealed on a PCR plate with a silicone gel cover to minimize evaporation during amplification using the ABI-PCR system. The Touch-Down amplification method included an initial pre-denaturation step at 94 °C for 3 min, followed by cycles of denaturation at 94 °C for 30 s, annealing starting at 65 °C for 45 s with a decrease of 0.7 °C per cycle for 13 cycles, and extension at 72 °C for 1 min. This was followed by 23 cycles of denaturation at 94 °C for 30 s, annealing at 56 °C for 45 s, and extension at 72 °C for 1 m, with a final extension at 72 °C for 5 min. This post-mixing centrifugation step was repeated for 30 s. The ILP amplification products were run on a 6% non-denaturing polyacrylamide gel, with each well supplemented with 2 µL of 10 × Loading Buffer, followed by a 30 s centrifugation to ensure proper loading.

### 4.4. Data Analysis

The electropherograms (Figure S1) generated from the experiments were interpreted manually. Clearly visible bands were assigned a value of “1”, while the absence of bands was denoted as “0”. This process produced raw data binary data (0 and 1) for subsequent analyses. Initial calculations and analyses of the electrophoretic bands were performed using Microsoft Excel. The binary data were then converted into genotypic data using DataFormater 2.7 software [61]. Genetic diversity analysis was analyzed using Popgene 1.31 software [62], which calculated several indices, including the number of observed alleles (Na), effective alleles (Ne), Shannon index (I), observed heterozygosity Ho), expected heterozygosity (He), Nei diversity index (H), genetic similarity coefficient (S), genetic distance (D), and gene flow (*N*m). Allele richness (*A*r) and private allele richness (*P*_A_) were calculated using the HP-RARE 1.1 software [63]. The nonparametric Kruskal–Wallis test was conducted in SPSS 25 to evaluate significant differences in Na, Ne, I, He, Ho, and *A*_R_ between the two tea tree populations. Allele frequencies derived from this analysis were used to calculate the primer polymorphic information content (PIC) of each primer using the PIC_CALC tool (version 0.6) [39]. Genetic variation within and between populations was assessed using the WINAMOVA v1.55 program. The significance of the variance components was evaluated using nonparametric permutation tests, with 1000 permutations performed for statistical validation [64].

The genetic structure of the population was assessed using Structure 2.3.4 [65], with the K value for population structure analysis ranging from 2 to 7. For each K value, the analysis was repeated ten times to improve reliability. A burn-in period of 50,000 was set, and Markov Chain Monte Carlo (MCMC) simulations were conducted for 100,000 iterations. The optimal K value was determined based on the likelihood value (Ln P(D)) and ΔK. Clustering was performed using MEGA11 (v. 11.0.13) based on genetic distance, and the Unweighted Pair Group Method with Arithmetic Averaging (UPGMA), and a dendrogram was constructed. Finally, PCoA was performed using GenALEx 6.503 software [66], which provided a graphical representation of the genetic relationships and variations observed in the dataset.

## 5. Conclusions

In conclusion, this study provides important insights into the genetic diversity and population structure of wild ancient *C. tetracocca* populations in Pu’an, Guizhou, China. The results indicate a high level of genetic diversity within the population, with Qingshan Township exhibiting greater genetic diversity than Xindian Township. This suggests that geographical factors play significant roles in shaping genetic diversity. These findings highlight the importance of conserving the genetic diversity of wild ancient *C. tetracocca* populations in Pu’an and offer scientific guidance for the development of targeted conservation strategies. Conservation efforts should prioritize the comprehensive protection of genetically diverse subpopulations, particularly in the Qingshan area, to ensure the long-term survival and sustainability of this unique and valuable species.

## Figures and Tables

**Table 1:** The genetic parameters of 40 ILP markers.

Primer	Na	Ne	I	Ho	He	H	PIC
Tea_ILP1116	6.00	3.99	1.51	0.00	0.75	0.75	0.75
Tea_ILP1418	4.00	2.50	1.01	0.89	0.60	0.60	0.60
Tea_ILP1396	4.00	2.42	0.99	0.22	0.59	0.59	0.59
Tea_ILP1000	5.00	2.87	1.27	0.00	0.65	0.65	0.65
Tea_ILP1589	3.00	2.21	0.93	0.39	0.55	0.55	0.55
Tea_ILP900	3.00	1.68	0.73	0.28	0.41	0.41	0.41
Tea_ILP1097	3.00	2.03	0.73	0.00	0.51	0.51	0.51
Tea_ILP1023	6.00	4.36	1.58	0.04	0.77	0.77	0.77
Tea_ILP1222	4.00	2.19	0.92	0.17	0.55	0.54	0.54
Tea_ILP1192	4.00	1.61	0.77	0.18	0.38	0.38	0.38
Tea_ILP1073	3.00	1.36	0.51	0.21	0.26	0.26	0.26
Tea_ILP591	7.00	5.49	1.81	0.00	0.82	0.82	0.82
Tea_ILP1158	5.00	4.26	1.51	0.48	0.77	0.77	0.77
Tea_ILP072	3.00	1.79	0.70	0.58	0.44	0.44	0.44
Tea_ILP015	4.00	3.50	1.31	0.00	0.72	0.71	0.71
Tea_ILP290	2.00	1.75	0.62	0.00	0.43	0.43	0.43
Tea_ILP380	6.00	4.35	1.59	0.39	0.77	0.77	0.77
Tea_ILP450	3.00	1.83	0.78	0.46	0.46	0.45	0.45
Tea_ILP202	4.00	2.62	1.07	0.00	0.62	0.62	0.62
Tea_ILP284	4.00	3.19	1.23	0.00	0.69	0.69	0.69
Tea_ILP1875	8.00	6.72	1.99	0.00	0.85	0.85	0.85
Tea_ILP1946	4.00	3.20	1.23	0.00	0.69	0.69	0.69
Tea_ILP1986	10.00	8.01	2.19	0.00	0.88	0.88	0.88
Tea_ILP2114	7.00	2.89	1.32	0.00	0.66	0.65	0.65
Tea_ILP2142	4.00	1.81	0.79	0.01	0.45	0.45	0.45
Tea_ILP2171	5.00	3.50	1.37	0.00	0.72	0.71	0.71
Tea_ILP1923	3.00	2.07	0.79	0.82	0.52	0.52	0.52
Tea_ILP1924	6.00	1.47	0.70	0.00	0.32	0.32	0.32
Tea_ILP1945	4.00	2.29	1.00	0.61	0.57	0.56	0.56
Tea_ILP1951	4.00	2.04	0.94	0.38	0.51	0.51	0.51
Tea_ILP1967	4.00	3.03	1.15	0.00	0.67	0.67	0.67
Tea_ILP1982	5.00	1.93	0.94	0.00	0.48	0.48	0.48
Tea_ILP1991	4.00	2.41	1.05	0.58	0.59	0.58	0.58
Tea_ILP2017	6.00	3.96	1.56	0.00	0.75	0.75	0.75
Tea_ILP2551	3.00	1.55	0.57	0.44	0.36	0.36	0.35
Tea_ILP3195	5.00	2.51	1.13	0.00	0.60	0.60	0.60
Tea_ILP1959	3.00	2.02	0.79	0.72	0.51	0.51	0.51
Tea_ILP3087	3.00	1.16	0.28	0.13	0.14	0.14	0.14
Tea_ILP1953	4.00	2.86	1.14	0.00	0.65	0.65	0.65
Tea_ILP2343	5.00	3.04	1.28	0.00	0.67	0.67	0.67
Mean	4.50	2.86	1.10	0.20	0.58	0.58	0.58

Note: the number of observed alleles (Na), the number of effective alleles (Ne), the Shannon information index (I), the observed heterozygosity (Ho), the expected heterozygosity (He), the Nei gene diversity index (H), the polymorphic information content (PIC).

**Table 2:** Genetic diversity analysis of wild ancient populations of *C. tetracocca* in Pu’an.

Population	Sample Size	Na	Ne	I	Ho	He	H	A_R_	P_A_
Qingshan town	90	4.38	2.98	1.13	0.20	0.60	0.60	2.54	0.89
Xindian town	48	4.05	2.30	0.90	0.20	0.50	0.50	2.21	0.57
*p* value		0.04 *	0.03 *	0.02 *	0.70	0.03 *	0.03 *	0.02 *	0.00 *

Note: the number of observed alleles (Na), the number of effective alleles (Ne), the Shannon information index (I), the observed heterozygosity (Ho), the expected heterozygosity (He), the Nei gene diversity index (H), Allelic richness (A_R_), Private allelic richness (P_A_), * indicate a significant difference at the 0.05 level.

**Table 3:** Genetic differentiation among wild ancient tea populations of *C. tetracocca* in Pu’an.

POPGENE				AMOVA				
*F*_is_	*F*_it_	*F*_st_	*N*_m_	Source of Variation	df	SS	var. Components	PMV%
0.64	0.65	0.04	6.85	Among population	1	519.24	1.93	8.41 (*p* < 0.001)
				Within populations	136	4631.68	21.02	91.59 (*p* < 0.001)

Note: inbreeding coefficient within a population, *F*_is_; total population inbreeding coefficient, *F*_it_; population differentiation coefficients, *F*_st_; population gene flow, *N*_m_; degrees of freedom, df; square deviation, SS; variance components, var. components; percentage of molecular variance, PMV.

**Table 4:** The information on wild ancient tea populations of *C. tetracocca*.

Population	Typology	Sample Size	Longitude	Latitude	Altitude (m)
Qingshan town	Wild-type	90	104.96–104.98	25.43–25.45	1691–1744
Xindian town	Wild-type	48	105.03–105.04	25.70–25.71	1822–1834

**Table 5:** A total of 40 Tea_ILP markers were used in the genetic diversity and population structure analysis of *C. tetracocca*.

Marker ID	Forward Primer 5′-3′	Reverse Primer 5′-3′
Tea_ILP1116	AGTAGGGTTTTTGCCTCCGT	AAGTACCGTACCCGCACTTG
Tea_ILP1418	ATTGCATTCTTCCTCGCACT	TCACAAATCAATTCCACCGA
Tea_ILP1396	CATCTCTCTCCTCCCTGCTG	TGTTCTATGGGATCTTCGCC
Tea_ILP1000	CCACCGGTTTCTGAAGATGT	CGCCTCCTTCTCTTTCCTCT
Tea_ILP1589	TCTGCTGCTAGATGCAAATGA	AGGAGCAACATAATTTGGCG
Tea_ILP900	GCTCGCACTCCAAGTAGGAT	TGACCTTGAAGCCAAAATTAAA
Tea_ILP1097	GCAGGAGACCTACTGGATGC	AAAGCGACAGTAGCCAGGAA
Tea_ILP1023	GGTGTAACCCAAGATCCCCT	TCCCACTGTCGATGTCTCAG
Tea_ILP1222	GCTGAGTTTCCTTTGGCAAG	CAAATGCATAATGTGGTCGC
Tea_ILP1192	GGCTGGGAATATGCTCTCAA	TTTGCAGAGCACTGAGGTTG
Tea_ILP1073	AATTCAGCCATCTGTCCAGC	TGACAGGATGGGCTTAAAGG
Tea_ILP591	TCATCGTTGTCGAGATTGGA	GACATGGCTGAGAGGAGAGG
Tea_ILP1158	CTTCGACTGAACACCCTCGT	GGAGGTGCAAACCACCTTTA
Tea_ILP072	TGCTTCATGTGCAGAACCAT	CTTGAACGACAACCCTCCAT
Tea_ILP015	AGATGCAGACGGAGAGCAAT	GCTTGCTTGGTTCAGGTAGC
Tea_ILP290	TTGAGATTCACTGCATAGCCA	AAATTCACTCAAACGGCCTG
Tea_ILP380	CTCGATAGGTTTGGGGTCAA	AATTGTGGGTGGCTCGTTAC
Tea_ILP450	TTGGGTTCGAAGAGTTGAGG	TAGCGAAAACGAAAGCCAGT
Tea_ILP202	TGAATCCGCCTTAACCAAAC	GAGCAACAACAGTTGGCTGA
Tea_ILP284	GAAATGAAACTGCCCATGCT	TAGGAGCAGGTGCTGGAACT
Tea_ILP1875	ACGACTCCAAGGTGGTTTTG	AAGGAAGCCCTTTCTGGATG
Tea_ILP1946	TTTCGCATTCTATCAAACATGG	TGATGGTATGACATGGTGCC
Tea_ILP1986	TTTCTTCCTCCACAGCAAGC	CTTGCGCACTTCTTCCTTGT
Tea_ILP2114	GATTTTGGCGCTCATCATTT	GCTTTTATCGTCCATCGGAA
Tea_ILP2142	CAAATTCCAACAACAATGCG	TTCCCCACCAAATTCGTTT
Tea_ILP2171	TGAAGTATGCCCACTTGGAA	TAAAGGATGCAGTTGGTCGG
Tea_ILP1923	GCTTCAAAGCCTATGCAAGAA	GGGAAGATCAGAGGCATTCA
Tea_ILP1924	GAGCACGGATTCAGTTGTCA	GAGCCACCCTTCCCAATTAT
Tea_ILP1945	AAGGCCATTGAGCACAAAAC	GGGGCAATAAATGACAATGG
Tea_ILP1951	AAGGCAATCAAGCAAGCACT	GAGGAAGCAGTTGCATCACA
Tea_ILP1967	TCTCGTGTTGTGGGCAAATA	TTGTTCAGGGCTCTTGCTTT
Tea_ILP1982	GCAAATGGAAAGCTTGTGCT	AATGCCATCCTCTCAAATGC
Tea_ILP1991	AAAGCTGGCAGGGTCATCT	GGCAAGGATGACAAGGCTAA
Tea_ILP2017	GATGTGCCGTGTTGTGAGAC	GCATTGCATATGAGGAGGGT
Tea_ILP2551	GGATTCCTCCTCAAACTCTTCA	TGCCAGCCTTCTTCTCTTTC
Tea_ILP3195	CATTACCAATGGCAAATCCC	CTCGAGTCCACCAAGGAAAC
Tea_ILP1959	ACACTGTGTCAGTTGGCGAG	TTCGAGATCGAATGTTTAGGC
Tea_ILP3087	GACCAGAAAATTGGGCATTG	AGCTGCATGTTCAGCAACAA
Tea_ILP1953	TATGCTGAAGCCCACACATC	TGTGGACCATGCAAGGTTAG
Tea_ILP2343	AAACCAGCGAGATGGAACAC	AGATCGACGGGATTGAGTTG

## Data Availability

All data were shown in Tables and Figures in the main text or Supplementary Materials.

## Supplementary Materials

The following supporting information can be downloaded at: https://www.mdpi.com/article/10.3390/plants14111709/s1, Figure S1: Detection of ILP marker amplification products of wild ancient tea tree population using PAGE gel electrophoresis.
