# Supplementary figures and images for: Genetic Diversity and Population Structure of Wild Ancient Camellia tetracocca in Pu’an, Guizhou, China
Source: Plants (Basel). 2025 Jun 4;14(11):1709. doi: 10.3390/plants14111709 (PMC12156966; doi:10.3390/plants14111709)

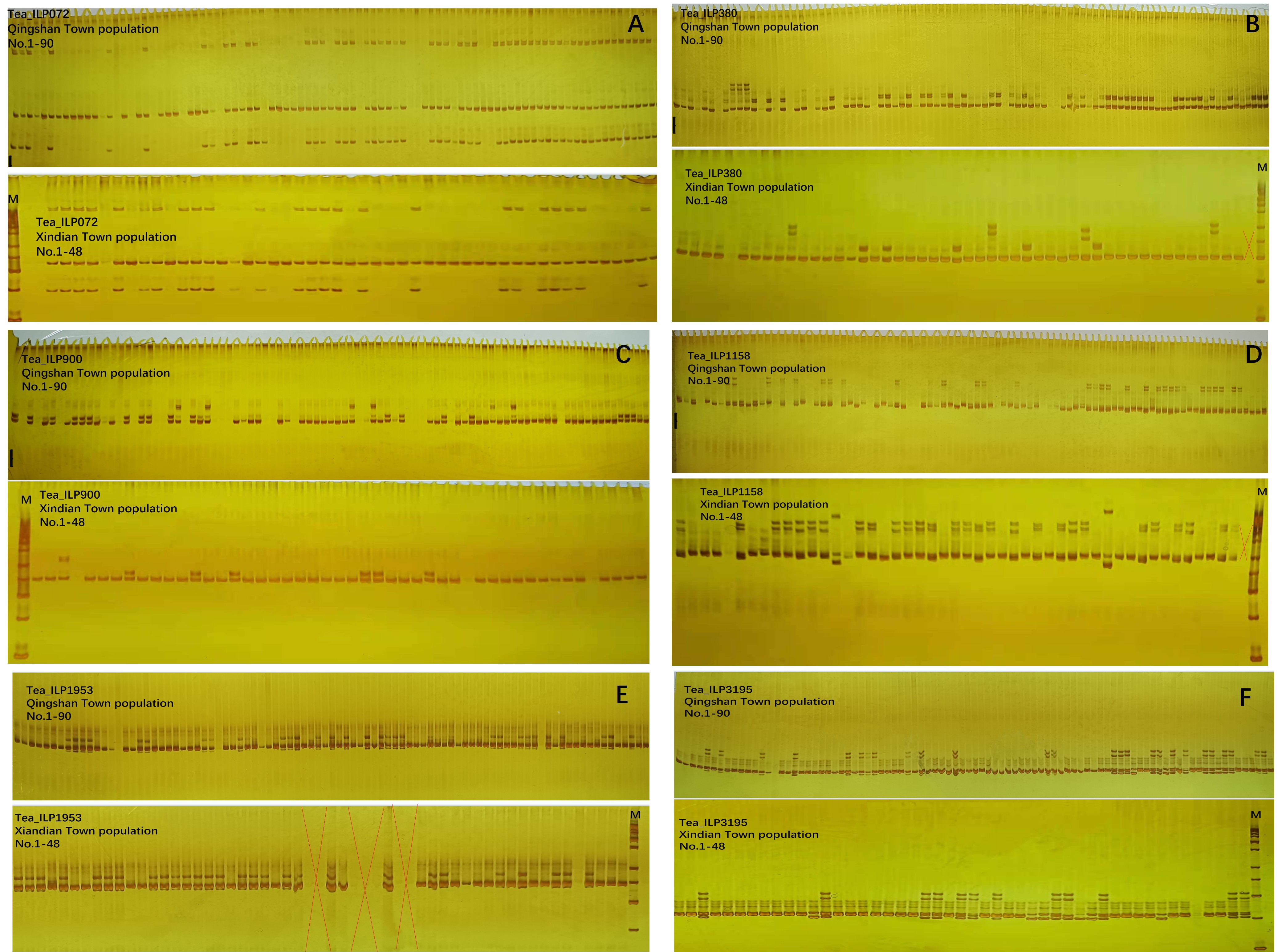

Supplement: Supplementary file 1 [file plants-14-01709-s001.zip › Figures_Supplementary 20250601/Figure S1.jpg]
